# Age-Friendly Universities: Confronting Ageism and Fostering Age Inclusivity

**DOI:** 10.1093/geroni/igaa057.1724

**Published:** 2020-12-16

**Authors:** Joann Montepare, Kimberly Farah

## Abstract

The pioneering Age-Friendly University (AFU) initiative, endorsed in 2016 by GSA’s Academy for Gerontology in Higher Education (AGHE), calls for institutions of higher education to respond to shifting demographics and the needs of aging populations through more age-friendly programs, practices, and partnerships. Over 65 institutions in the United States, Canada, European countries, and beyond have joined the network and adopted the 10 AFU principles. Despite the importance and appeal of the AFU initiative, individuals leading age-friendly efforts on their campuses have found that ageism in higher education is a persistent, yet overlooked, factor holding us back from embracing age diversity. This symposium will feature AFU partners who will discuss how ageism presents itself in higher education, along with offering recommendations for breaking it down and promoting greater age inclusivity. Montepare will open the session with an overview of systematic and implicit instances of ageism in higher education. Bowen and colleagues will then discuss results from an AFU Campus Climate Survey that examined the age attitudes of faculty, students, and staff along with their views about that nature of campus age-friendliness. Manoogian will discuss the value of approaching the teaching of age diversity from an intersectionality perspective. Reynolds and Kruger will provide theoretical framing and dissemination models for the GSA online course Ageism First Aid within various AFU and programmatic structures. Andreoletti and June will discuss how creating an age-inclusive AFU Learning Community can raise awareness about ageism across campus as well as in the community where a campus resides. Age-Friendly University (AFU) Interest Group Sponsored Symposium.

